# Efficient Load-Bearing Capacity Assessment of a Degraded Concrete Manhole Using Sectional Homogenization

**DOI:** 10.3390/ma17235883

**Published:** 2024-11-30

**Authors:** Tomasz Garbowski, Tomasz Grzegorz Pawlak, Anna Szymczak-Graczyk

**Affiliations:** 1 Department of Biosystems Engineering, Poznan University of Life Sciences, Wojska Polskiego 50, 60-627 Poznan, Poland; tomasz.garbowski@up.poznan.pl; 2 Department of Construction and Geoengineering, Poznan University of Life Sciences, Piątkowska 94E, 60-649 Poznan, Poland; tomasz.pawlak1@up.poznan.pl

**Keywords:** sectional homogenization, concrete repair, three-layer protective coating, polyurethane, polyurea, sulfate corrosion, mechanical strength restoration

## Abstract

This study addresses a practical and efficient approach to evaluating the load-bearing capacity of severely degraded concrete manholes. Concrete deterioration, often advanced and highly irregular, can be captured accurately through surface scanning to create a detailed model of the damaged structure and also to build a simplified modeling to enable rapid engineering-level assessment, filling a critical gap in infrastructure maintenance. The repair strategy involves applying an internal polyurea layer, a variable-thickness polyurethane foam layer depending on the degree of localized degradation, and an external polyurea layer to restore the original shape of the manhole. However, these repairs do not fully restore the manhole’s original load-bearing capacity. A full 3D model, encompassing millions of finite elements, would provide a detailed analysis of strength reductions but is impractical for engineering applications due to computational demands. An alternative approach utilizing sectional homogenization is proposed, where sectional properties are sequentially averaged to calculate effective parameters. This approach enables the use of only a few hundred shell elements, each representing thousands of elements from the detailed 3D model, thus providing a rapid, engineering-level assessment of load-bearing reductions in degraded manholes. The study finds that while the repair method restores up to 76% of bending stiffness in heavily corroded sections, it does not fully recover the original load-bearing capacity.

## 1. Introduction

Corrosion, especially sulfate and microbiologically induced corrosion, poses significant threats to sewer infrastructure because it weakens concrete structures, leading to premature degradation [1,2,3,4,5]. Sulfate corrosion in sewage networks is an increasingly recognized problem [3,4,5], with studies showing that acidic environments created by sulfur bacteria play a critical role [2,6]. Grengg et al. [7] provided a detailed case study on microbiological corrosion in a combined sewer network, while Jiang et al. [6] used artificial neural networks to predict sewer corrosion. Jeyapalan [8] offers insights into mitigating sulfide-induced corrosion, an approach particularly relevant for long-term infrastructure maintenance.

Concrete protection techniques focus on specialized coatings and additives to counteract aggressive chemical and environmental conditions [9,10,11,12,13]. Protective measures such as cementitious coatings are shown to effectively shield concrete from harmful gases like H_2_S [14,15]. O’Connell et al. [16] discuss biochemical attacks in wastewater applications, emphasizing the efficacy of antimicrobial compounds and other surface treatments [11]. Wang et al. [17] investigated fast-curing repair concretes around manholes, contributing to prolonged concrete durability in aggressive environments.

Recent advancements in repair technologies, such as polyurea and polyurethane, show promise in enhancing damaged concrete structures [18,19,20,21,22]. Szafran and Matusiak [13,21] explored polyurea coating systems, finding that these materials improve both chemical resistance and structural integrity. Awassa et al. [9] presented findings on strengthening underground concrete structures using carbon fiber sheets, significantly enhancing load-bearing capacity. Similarly, Davidson et al. [23] studied polymer-reinforced walls exposed to blast effects, noting improvements in resilience.

Various methods are employed to protect concrete structures in water and wastewater infrastructure, particularly those exposed to aggressive chemical environments such as sulfate corrosion. Traditional approaches include the use of cement-based mortars, resins, glass-reinforced plastic (GRP) liners, and polymer concrete modules. Cement-based mortars are often used for their structural reinforcement capabilities but can suffer from porosity and limited chemical resistance. Resin coatings, such as polyurethane and epoxy, provide a higher degree of chemical resistance, but their effectiveness can be limited by application inconsistencies and the presence of seams or joints. GRP liners and polymer concrete modules offer structural reinforcement but often require labor-intensive installation processes and have limitations regarding the complete sealing of treated surfaces [24].

In recent years, innovative solutions have been developed to address these challenges, including the use of polyurea-based coatings. The article at hand discusses a novel three-layer polyurea coating system, which significantly advances the state of repair and protection for infrastructure elements exposed to highly aggressive environments. The system comprises a moisture-blocking polyurea base layer, a middle layer of rigid polyurethane for structural reinforcement, and a final sealing layer of polyurea. This approach creates a seamless, monolithic protective structure, eliminating the risks associated with joints and seams found in traditional methods. Additionally, the rapid curing time of polyurea minimizes downtime and allows for quick restoration of operational use, making it a highly efficient option for infrastructure maintenance and protection [24].

The development of new materials, like hybrid fiber-reinforced geopolymers (HFRG), offers alternatives for repairing and reinforcing aging structures [8,25,26]. These materials have shown promising results in preventing environmental damage to concrete and masonry [8,25]. Guades et al. [25] explored the application of HFRG in seismic rehabilitation, while Johnstone [15] reported on the benefits of using cementitious coatings to renew old sewer infrastructure.

Homogenization and optimization techniques are essential in analyzing thin-walled structures and concrete slabs, improving the efficient use of materials [16,27]. Gajewski, Staszak, and Garbowski [2,18] introduced optimization strategies for thin-walled beams with perforations, highlighting the benefits of material efficiency. Staszak et al. [26,27,28] examined the effective stiffness of concrete slabs using numerical homogenization, which facilitates accurate prediction of structural behavior. These methodologies are key for optimizing structural design and enhancing durability in repaired sections.

In extreme conditions, such as exposure to blast or high temperatures, concrete requires specific damage prevention strategies [20,23,29,30,31]. Davidson et al. [23] analyzed blast-resilient, polymer-reinforced walls, while Parniani and Toutanji [32] examined fatigue performance in polyurea-coated RC beams, finding that specialized coatings significantly improve resilience. Similarly, Buczkowski’s studies [33,34] focus on reinforcing slabs subject to thermal loads, addressing the structural requirements under extreme environmental stress.

Concrete corrosion, especially in sewer environments, poses significant challenges for maintaining the structural integrity of infrastructure. Traditional repair methods often fail to fully restore the mechanical properties of degraded structures. Recent advances in materials science, however, provide new opportunities to address these issues. For example, Wu et al. [35] demonstrated that incorporating recycled concrete and paste powder into engineered geopolymer composites (EGC) significantly improves their mechanical properties, particularly under tensile loads. Similarly, Wang et al. [36] highlighted the benefits of substituting recycled fine aggregates for traditional silica sand, enhancing both tensile strength and strain capacity through refined microstructure and active alkaline content. These studies underline the potential of innovative material solutions in restoring and even improving the performance of damaged concrete.

Building on these concepts, the present study adopts a practical approach focused on sectional homogenization and advanced composite coatings. This method leverages detailed 3D surface scanning and numerical modeling to restore load-bearing capacity efficiently. The integration of recent findings from material innovations into practical engineering applications ensures that repair strategies not only address existing damage but also enhance long-term durability under aggressive environmental conditions.

Accurate prediction and modelling are crucial for effective maintenance of aging infrastructure, particularly in assessing internal forces and optimizing tank designs [12,31,37,38,39,40,41]. Szymczak-Graczyk [30] and Garbowski et al. [12] demonstrated optimization techniques for the internal forces in tank walls, using advanced simulation methods to maximize material efficiency. MATLAB version 9.14.0 (R2023b) [19] software is frequently applied in such structural analyses, providing essential computational support.

Green alternatives like modified starches and dextrins are gaining traction as sustainable cement modifiers [20,42]. Sybis and Konował [20] discussed starch admixtures in cement composites, showing improvement in physicochemical properties. Similarly, Sybis, Konował, and Prochaska [42] explored biodegradable dextrins, noting their environmental benefits and potential for enhancing the sustainability of concrete repair methods.

In this study, a simplified yet effective method for assessing the load-bearing capacity of degraded concrete manholes was developed. Traditional full-scale 3D finite element models, while theoretically capable of simulating the structural response of such degraded structures, are computationally prohibitive. For instance, discretizing a 100 × 100 mm^2^ surface with 2 × 2 × 2 mm^3^ elements would require approximately 125,000 elements. By contrast, the proposed sectional homogenization approach reduces this complexity, enabling the same area to be effectively represented by just four 50 × 50 mm^2^ shell elements, achieving a reduction ratio of 1:32,250.

This methodology significantly reduces computational demands while maintaining accuracy in predicting load-bearing capacity. It accelerates the assessment process, making it practical for evaluating infrastructure with varying degrees of degradation, and offers a reliable solution for engineering-level applications.

## 2. Materials and Methods

### 2.1. Properties of Repaired Manholes

The composite protective system applied to the degraded sewer manholes is composed of three distinct layers designed to enhance both the chemical resistance and structural integrity of the damaged sections:

1. Concrete Base Layer: Serving as the primary load-bearing substrate, this layer has a Young’s modulus of E = 30,000 MPa and a Poisson’s ratio of ν = 0.2, reflecting the mechanical properties of concrete commonly used in sewer infrastructure. This base layer is the most structurally significant in terms of supporting loads and providing overall rigidity to the composite system.
2. Polyurea Coating Inner Layer: The inner polyurea layer functions as a chemical barrier, shielding the concrete from further environmental degradation. Inner polyurea layer has very low stiffness properties, with a Young’s modulus of E = 100 MPa and Poisson’s ratio ν = 0.35.
3. Middle Layer—Polyurethane Foam: A closed-cell polyurethane foam layer, characterized by a Young’s modulus E = 800 MPa and Poisson’s ratio ν = 0.05, is employed to reconstruct the geometry of the corroded or damaged sections. Although the foam does not add significant stiffness, it contributes to restoring the original shape and volume of the concrete section.
4. Polyurea Coating Outer Layer: The outer polyurea layer functions as a chemical barrier, shielding the foam from further environmental degradation. Outer polyurea layer has same stiffness properties as inner one, with a Young’s modulus of E = 100 MPa and Poisson’s ratio ν = 0.35.

Both polyurea layers are responsible for enhancing the chemical resilience of the entire protective system.

Polyurethane foam and polyurea coatings each exhibit material properties that make them ideal for rehabilitating degraded infrastructure, with specific numerical ranges highlighting their capabilities. Polyurethane foam is characterized by a low density, typically ranging from 30 to 60 kg/m^3^, making it lightweight and ideal for minimizing additional structural loads. Its compressive strength generally falls between 200 and 800 kPa, providing sufficient mechanical support for void filling and load distribution. Additionally, its closed-cell structure offers water absorption rates below 2%, ensuring minimal moisture penetration, while its thermal conductivity ranges from 0.02 to 0.03 W/(m·K), making it highly effective as an insulator.

Polyurea coatings, on the other hand, are recognized for their flexibility and mechanical resilience. Their tensile strength typically ranges from 15 to 30 MPa, with elongation at break often exceeding 300%, allowing them to accommodate significant structural movements without cracking. Polyurea also exhibits excellent chemical resistance, with impermeability to water and a water vapor transmission rate of less than 1 g/m^2^ per day. It remains stable across a wide temperature range, from −40 °C to 120 °C, and demonstrates outstanding abrasion resistance, with a Taber wear index below 50 mg/1000 cycles (depending on formulation). These numerical properties underscore the suitability of polyurethane foam and polyurea in creating a durable and effective protective system for infrastructure exposed to aggressive environmental conditions.

**Figure 1:**
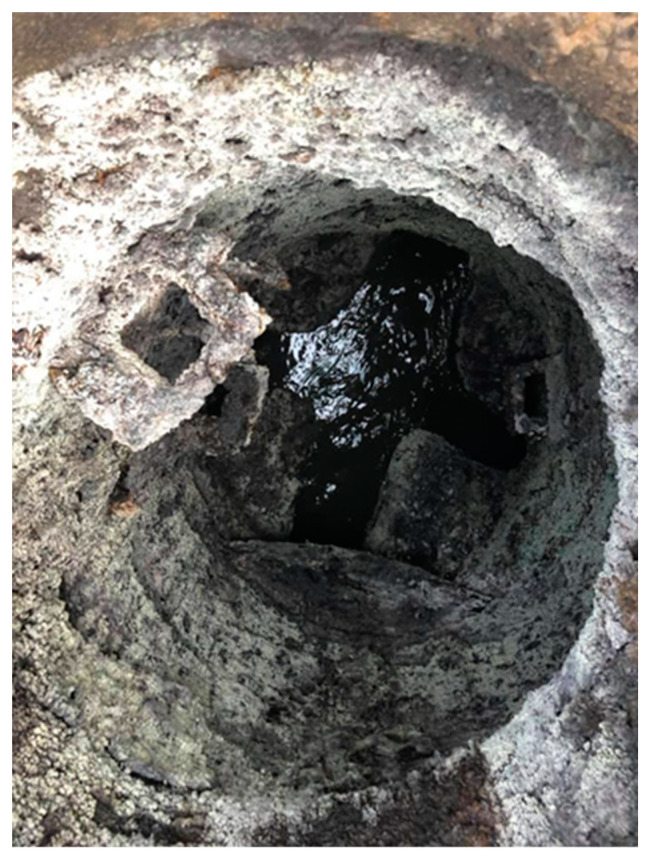
Heavily corroded manhole.

Concrete corrosion in sewer infrastructure is predominantly caused by sulfate and microbiologically induced corrosion, which severely degrade the structural integrity of the material. Sulfate ions react with calcium hydroxide and other compounds in the cement matrix, forming expansive products such as gypsum and ettringite. This leads to cracking, spalling, and significant loss of cross-sectional area, as shown in Figure 1. Additionally, microbiologically induced corrosion involves sulfur-oxidizing bacteria that generate sulfuric acid, further accelerating the degradation process. The characteristic surface irregularities and deep pitting resulting from these processes can be also observed, highlighting the challenges in accurately assessing and repairing such damage.

### 2.2. Assembly of Stiffness Matrix for Finite Element Models

In this study, a 3D finite element model (FEM) is developed to analyze the structural response of degraded concrete manholes using eight-node hexahedral elements. This approach allows for detailed modeling of the degradation patterns and repair layers. Additionally, a simplified model is employed using four-node shell elements to represent the homogenized cross-section, reducing computational complexity while preserving core mechanical behaviors.

#### 2.2.1. Element Selection

In the 3D detailed model, eight-node hexahedral elements are chosen to capture the three-dimensional stress and strain distributions accurately, accounting for degradation and repair layers. In the simplified model, four-node shell elements are used to approximate the response of the homogenized cross-section, significantly reducing the number of elements and thus computational requirements. This combination allows the model to balance accuracy with efficiency, using the 3D elements for detailed analysis and shell elements for broader structural evaluation. 

#### 2.2.2. Integration Scheme

For each hexahedral element in the 3D model, the stiffness matrix $K_{e}$ is computed using Gaussian quadrature with eight integration points, allowing precise capture of stiffness contributions across the three-dimensional structure. The integration scheme for shell elements in the simplified model also uses Gaussian quadrature, adjusted to the two-dimensional nature of these elements, to compute effective stiffness properties. The stiffness matrix $K_{e}$ for both element types is given by:

(1) $$K_{e}=\int_{v}\;B_{s}^{T}D_{m}B_{s}dV,$$

where $B_{s}$ is the strain-displacement matrix, relating element displacements to strains, D is the material property matrix, defined by the Young’s modulus E and Poisson’s ratio ν for each layer, and V represents the volume of the element.

#### 2.2.3. Strain-Displacement Matrix $B_{s}$

In the 3D model, the strain-displacement matrix $B_{s}$ relates nodal displacements to strains, capturing the complex three-dimensional strain variations due to irregular degradation. For shell elements in the simplified model, the $B_{s}$ matrix accounts for averaged cross-sectional strains, assuming uniform strain distribution through the shell thickness.

#### 2.2.4. Shape Functions for Eight-Node Hexahedral and Four-Node Shell Elements

For eight-node hexahedral elements, the shape functions are defined in natural coordinates (ξ,η,ζ), varying from −1 to 1 for each direction, to facilitate integration and capture volumetric deformations. In the simplified model, four-node shell elements employ bilinear shape functions based on $\left(\xi ,\eta \right)$ for interpolation across the element, focusing on planar deformation.

#### 2.2.5. Material Property Matrix $D_{m}$

The $D_{m}$ matrix represents material stiffness under both 3D and 2D (shell) conditions. In the 3D model, $D_{m}$ reflects the isotropic elastic properties for each layer—concrete, polyurethane foam, and polyurea—while in the simplified shell model, $D_{m}$ represents the homogenized material properties computed from equivalent mechanical behavior. The material matrix $D_{m}$ for the composite shell element accounts for the combined effect of each layer’s properties to represent the repaired section effectively.

In the 3D model, each hexahedral element assumes an isotropic, linearly elastic material, meaning that the relationship between stresses and strains is described by the material stiffness matrix $D_{m}$ in three dimensions. The $D_{m}$ matrix has the following form:

(2) $$D_{m}=\frac{E\left(1+\nu \right)}{1-2\nu }\left[\begin{array}{cccccc} 1-\nu & \nu & v & 0 & 0 & 0\\ \nu & 1-\nu & v & 0 & 0 & 0\\ v & v & 1-v & 0 & 0 & 0\\ 0 & 0 & 0 & \frac{1-2\nu }{2} & 0 & 0\\ 0 & 0 & 0 & 0 & \frac{1-2\nu }{2} & 0\\ 0 & 0 & 0 & 0 & 0 & \frac{1-2\nu }{2} \end{array}\right].$$

The elements of the $D_{m}$ matrix in the 3D model describe material stiffness properties in all three main directions and shear moduli. The diagonal terms represent direct elastic properties in the x, y, and z directions, while the off-diagonal terms account for Poisson’s effects. The lower right corner entries correspond to the shear response in the xy, yz, and zx planes.

In the simplified shell model, based on cross-sectional homogenization, the material is also considered isotropic and linearly elastic but under axisymmetric conditions. Here, stresses and strains are considered in two dimensions (the cross-sectional plane), and the $D_{m}$ matrix is simplified as:

(3) $$D_{m}=\frac{E}{1-\nu^{2}}\left[\begin{array}{ccc} 1 & \nu & 0\\ \nu & 1 & 0\\ 0 & 0 & \frac{1-\nu }{2} \end{array}\right].$$

In this matrix, the diagonal terms represent stiffness in the radial and axial directions, while the lower right term corresponds to the shear stiffness in the plane of the shell. The off-diagonal terms represent Poisson effects in the plane, capturing transverse deformations.

### 2.3. Homogenization Process

The objective of the homogenization process is to simplify the multi-layered structure of a degraded concrete manhole, including its repair layers, by representing it as a single homogeneous layer with effective mechanical properties. This transformation leverages strain energy equivalence, allowing the simplified model to capture the structural response of the original complex system without requiring an extensive computational setup.

#### 2.3.1. Static Condensation and Stiffness Matrix Reduction

The process begins by constructing a representative volume element (RVE) of the structure, which is used to capture the mechanical properties of the full 3D system. For each RVE, a global stiffness matrix K is assembled and then condensed to focus on the external nodes (primary degrees of freedom) by eliminating internal nodes. This results in a condensed stiffness matrix $K_{ext}$, calculated as:

(4) $$K_{ext}=K_{ee}-K_{ei}K_{ii}^{-1}K_{ie},$$

where $K_{ee}$ and $K_{ii}$ correspond to external and internal nodes, respectively, within the RVE, and $K_{ei}$ and $K_{ie}$ link internal nodes to external nodes.

This condensation reduces the complexity of the model while retaining the essential structural response characteristics of the full RVE model.

#### 2.3.2. Energy Equivalence and Transformation

The homogenization approach relies on the principle of strain energy equivalence between the full 3D RVE model and the simplified single-layer shell model. Displacements at the RVE’s external nodes are set to trigger both membrane and bending behavior, with generalized strains mapped to boundary displacements using a transformation matrix A:

(5) u=Aϵ,

where u is the displacement vector and ε is the strain vector.

This transformation matrix A links the positions of external nodes on the RVE boundary with generalized constant strains, allowing the simplified shell model to represent the behavior of the original multi-layered structure.

#### 2.3.3. Relationship Between Displacements and Effective Strains

The transformation from the full model to the homogenized model starts by defining the relationship between displacements and generalized strains at the boundary nodes. For a single node, this relationship is described as:

(6) $$u_{i}=A_{i}\epsilon_{i},$$

where $u_{i}$ is the displacement vector for node i, $\epsilon_{i}$ is the strain vector, and $A_{i}$ is a matrix relating displacements to effective strains at the boundary nodes.

The **A** matrix for a solid RVE model, capturing both membrane and bending behaviors, is expressed as [18,28]:

(7) $$A_{i}=\left[\begin{array}{cccccccc} x & 0 & y/2 & z/2 & 0 & xz & 0 & yz/2\\ 0 & y & x/2 & 0 & z/2 & 0 & yz & xz/2\\ 0 & 0 & 0 & x/2 & y/2 & -x^{2}/2 & -y^{2}/2 & -xy/2 \end{array}\right].$$

This matrix $A_{i}$ defines the effective strain distribution across the RVE boundary nodes, facilitating the condensation of the stiffness matrix.

#### 2.3.4. Elastic Strain Energy Equivalence

The total elastic strain energy E in the system, essential for energy equivalence between the full and simplified models, is defined by:

(8) $$E=\frac{1}{2}u_{e}^{T}K\;u_{e}=\frac{1}{2}\epsilon_{e}^{T}A_{e}^{T}KA_{e}\epsilon_{e}$$

where $u_{e}$ is the displacement vector for external nodes, K is the condensed stiffness matrix, and $A_{e}$ is the transformation matrix relating boundary node positions to generalized strains.

This energy equation ensures that the homogenized model has an equivalent elastic response to the full model under applied loads.

#### 2.3.5. Stiffness Matrix for Homogenized Composite Model

The stiffness matrix $A_{k}$ for the homogenized composite model is derived as follows [18,28]:

(9) $$A_{k}=\frac{A_{e}^{T}KA_{e}}{area}.$$

This condensed stiffness matrix $A_{k}$ allows the simplified shell model to effectively capture the bending and membrane behavior of the original multi-layer structure by using averaged properties.

#### 2.3.6. ABD Matrix and Effective Material Properties

The following matrices are utilized in determining the effective thickness and mechanical properties of the homogenized layer:

(10) $$D=\left[\begin{array}{ccc} D_{11} & D_{12} & 0\\ D_{21} & D_{22} & 0\\ 0 & 0 & D_{33} \end{array}\right];\;\;\;B=\left[\begin{array}{ccc} B_{11} & B_{12} & 0\\ B_{21} & B_{22} & 0\\ 0 & 0 & B_{33} \end{array}\right];\;\;\;A=\left[\begin{array}{ccc} A_{11} & A_{12} & 0\\ A_{21} & A_{22} & 0\\ 0 & 0 & A_{33} \end{array}\right].$$

These matrices represent the bending stiffness (D), coupling (B), and membrane stiffness (A) of the multi-layer composite, enabling the homogenized layer to maintain an equivalent structural response to the full model [43,44,45].

#### 2.3.7. Effective Thickness Calculation

The effective thickness t for the homogenized shell model is calculated to minimize bending effects and is determined by [18,28]:

(11) $$t=\sqrt{12\;\frac{trace(D^{*})}{trace(A)}},$$

where $D^{*}=D-BA^{-1}B$ represents the modified stiffness matrix considering bending stiffness. This equation ensures that the thickness t accurately represents the multi-layered structure’s overall stiffness in the homogenized model.

#### 2.3.8. Computation of Effective Material Properties

Using the effective thickness t, the bending stiffness D, and membrane stiffness A, the following properties are determined for the orthotropic shell model [18,28]:

(12) $$E_{11}^{D}=\frac{12}{t^{3}}D_{11},\;\;\;E_{22}^{D}=\frac{12}{t^{3}}D_{22},\;\;\;G_{12}^{D}=\frac{12}{t^{3}}D_{33},\;\;\;\nu_{12}^{D}=\frac{D_{12}}{D_{22}}$$

and for membrane stiffness:

(13) $$E_{11}^{A}=\frac{1}{t}A_{11},\;\;\;E_{22}^{A}=\frac{1}{t}A_{22},\;\;\;G_{12}^{A}=\frac{1}{t}A_{33},\;\;\;\nu_{12}^{A}=\frac{A_{12}}{A_{22}}$$

These calculations yield effective material constants for the equivalent shell model, allowing the homogenized layer to accurately replicate the stiffness of the original structure.

#### 2.3.9. Transverse Shear Stiffness

The transverse shear stiffness $G_{13}$ and $G_{23}$ are derived from the R matrix components as follows:

(14) $$G_{13}=\frac{R_{11}}{kt},\;\;\;G_{23}=\frac{R_{23}}{kt},$$

where k is a shear correction factor and

(15) $$R=\left[\begin{array}{cc} R_{11} & 0\\ 0 & R_{22} \end{array}\right]$$

represents the transverse shear stiffness within the RVE, ensuring that shear effects are also captured in the homogenized model.

### 2.4. Stochastic Generation of the 3D RVE Model

The 3D RVE model of the degraded concrete manhole is designed to reflect the realistic shape of its surface, which, in practice, would be obtained from detailed surface scanning. Such scanning captures the complex and irregular degradation patterns typical in corroded structures.

In this study, to simulate the surface degradation, random material loss values are generated at 49 discrete points across the RVE. These values represent varying degrees of degradation. To create a continuous and realistic surface, a 2D Gaussian interpolation function is applied to these points. This Gaussian-based interpolation smooths the initial random values, creating a degraded surface profile that mimics the irregular, eroded shape of the actual manhole.

By using Gaussian interpolation, the model achieves a natural-looking transition between degraded and less degraded regions, effectively representing the complex surface characteristics without the need for high-resolution scan data. This approach provides a practical method for generating an RVE that can be used to analyze structural performance under realistic degradation conditions.

## 3. Results

As an illustrative example, let’s examine a concrete manhole with a diameter of 200 cm and an initial wall thickness of 120 mm that has suffered significant corrosion, resulting in material losses of up to several tens of percent. The repair process involved a three-layer protective and reinforcing coating, designed to restore the structural integrity of the manhole while compensating for the lost material.

The repair strategy was based on precise measurements of the degraded areas, obtained using a 3D scanner that captured the irregular surface profile caused by corrosion. This detailed scan allowed for an accurate assessment of the losses and informed the preparation of the surface for the application of the repair layers. The repair coating was applied in three stages:

- An internal polyurea layer for moisture protection,
- A polyurethane foam layer with variable thickness, tailored to match the depth of material losses, thereby both compensating for the degradation and increasing the cross-section by an additional 80 mm, and
- An external polyurea layer to enhance durability and protect against further environmental damage.

**Figure 2:**
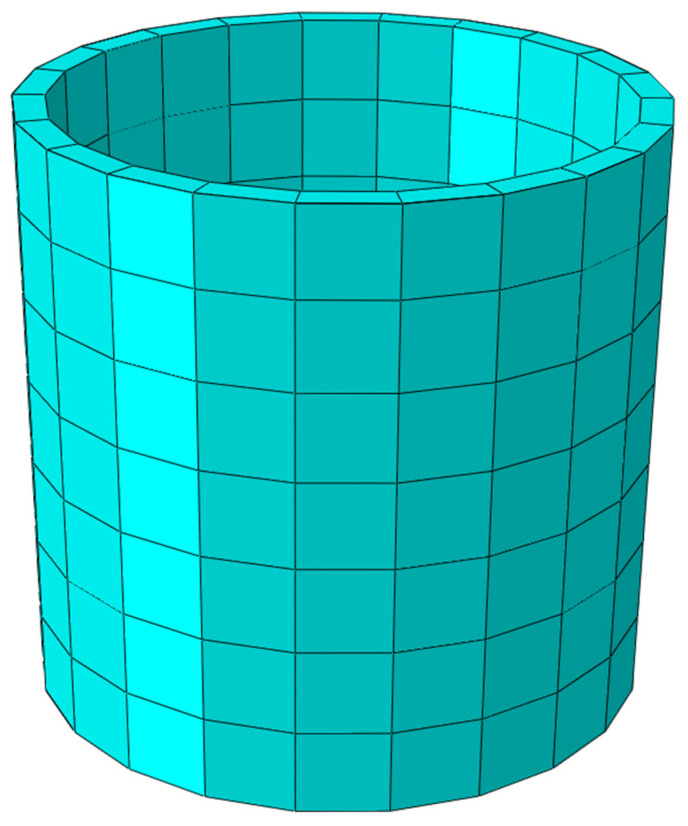
Homogenized shell model of the repaired manhole, with homogenization applied in each visible segment.

**Figure 3:**
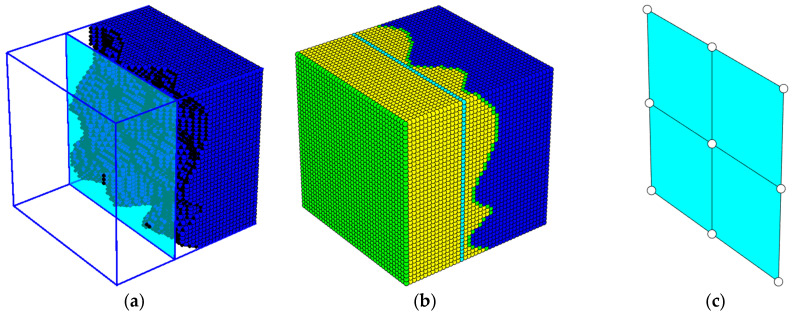
Representation of the 3D model and homogenized section of the corroded manhole segment. (**a**) A 3D model of the degraded concrete segment with detailed mesh capturing irregular corrosion patterns; (**b**) the repaired segment, showing the three-layer protective coating applied to the degraded area, with color-coded layers representing different materials; (**c**) a homogenized cross-section of the repaired segment, represented as shell elements for simplified simulation.

The resulting structure, with its adjusted cross-sectional profile, is depicted in Figure 2. In the 3D model, thousands of hexahedral finite elements are used to represent the original, degraded concrete structure (Figure 3a) and the applied repair layers in full detail (Figure 3b). Each shell finite element in the manhole model (shown in Figure 2) captures the specific thickness variations and material properties at different locations, providing a highly accurate representation of the repaired manhole.

Color Key for Figure 3b: dark blue—original concrete material in the manhole, showing areas affected by corrosion; cyan—areas where the concrete retained its original, non-corroded thickness; green—polyurea layer applied as a protective coating on the inner and outer surfaces; yellow—polyurethane foam layer, used to fill in material losses and provide additional reinforcement in the repaired section.

For computational efficiency, the complex 3D model is then homogenized, reducing the cross-section into a single, equivalent layer that can be simulated using shell elements (see Figure 3c). The homogenization process involves averaging the properties of the multi-layer cross-section, resulting in a simplified model that retains the essential mechanical characteristics of the original repaired structure but significantly reduces computational demands.

In the homogenized model, each shell element represents a region that, in the detailed 3D model, would contain thousands of individual hexahedral elements. This simplification enables a balance between accuracy and computational efficiency, allowing for effective simulations of the manhole’s structural behavior under load. The final homogenized shell model is shown in Figure 3c, where the effective single-layer representation demonstrates how the foam layer and polyurea coatings collectively restore the shape and approximate the mechanical response of the original structure.

This approach illustrates a practical application of 3D scanning and advanced finite element modeling in infrastructure repair, offering a method to efficiently simulate and assess the structural performance of heavily corroded concrete manholes after repair.

The changes in stiffness values due to degradation and repair are summarized in Table 1. This table compares the relative differences in the A, D, and R stiffness matrices for the least corroded segment, where the concrete volume loss was 10.19%. The columns show the changes in stiffness compared to the original manhole, the corroded manhole, and the repaired vs. corroded manhole, providing insight into how the repair method restores structural properties.

Table 2 contains the effective material parameters for the homogenized repaired section with an effective thickness of $t_{eff}=111.27$ mm. Using the effective thickness allows for the calculation of material parameters that ensure accurate analysis in both compressive states, where the **A** matrix is applied, and bending states, where the **D** matrix is used. More details on stiffnesses **A**, **D** and **R** are available in the Appendix A. This approach enables a consistent representation of the repaired structure’s behavior across different loading conditions. The incompatibility of parameters calculated using the total thickness of 200 mm is demonstrated by the values shown in parentheses, highlighting that only the effective thickness provides accurate material properties for reliable analysis.

In Table 3 and Table 4, the same analyses are presented as in Table 1 and Table 2, but for a more heavily corroded segment where the volume loss reached 38.27%. This set of tables illustrates the degradation’s impact on stiffness values and the effectiveness of the repair method for sections with more extensive material loss.

Each table contributes to understanding the progression of stiffness degradation and restoration, supporting the evaluation of the three-layer repair coating’s performance across different levels of corrosion.

Table 4 provides the effective material parameters for the heavily corroded and repaired segment with an effective thickness of $t_{eff}=80.92$ mm. Similar to Table 4, only the use of the effective thickness enables the determination of material parameters that ensure a correct analysis in compression (utilizing the **A** matrix) as well as in bending (utilizing the **D** matrix). The values in parentheses, calculated with the total thickness of 200 mm, further demonstrate the lack of compatibility in parameters obtained without using the effective thickness, underscoring the importance of homogenization for accurate structural representation.

The results demonstrate that both homogenization and segmentation are essential for accurately assessing the structural performance of repaired corroded manhole sections. Using the effective thickness in the homogenized model provides compatible material parameters for analysis in both compressive and bending states, whereas values calculated with the total thickness lack this compatibility, emphasizing the importance of these techniques for reliable performance evaluation.

## 4. Discussion

The analysis of the repaired corroded manhole sections reveals both the strengths and limitations of the applied three-layer protective coating. Although the repair method significantly improves the structural properties of the degraded concrete sections, certain stiffness reductions persist, demonstrating the inherent limitations of the approach in fully restoring the original mechanical performance.

The results, particularly from Table 1 and Table 3, highlight specific reductions in the **A**, **D**, and **R** matrices, which represent membrane, bending, and transverse shear stiffness, respectively. For the segment with a 10.19% volume loss, even after repair, the membrane stiffness Δ**A** is still reduced by approximately 8.45% compared to the original section, while the bending stiffness Δ**D** shows a reduction of about 7.93%. Similarly, the transverse shear stiffness Δ**R** remains 7.99% lower than in the original undamaged structure. These results indicate that, although the repair improves the structural integrity, it does not entirely compensate for the initial damage, particularly in areas where the concrete volume loss is substantial.

In the more heavily corroded section, where volume loss reaches 38.27%, the stiffness reductions are more pronounced. Post-repair values show a 35.82% reduction in membrane stiffness Δ**A**, a 50.60% reduction in bending stiffness Δ**D**, and a 35.17% reduction in transverse shear stiffness Δ**R**. These significant residual stiffness losses demonstrate that while the repair process can mitigate damage to a certain extent, it cannot fully restore the structure’s original strength and load-bearing capacity in cases of extensive corrosion.

Beyond mechanical performance, the polyurea-based coating system offers several advantages relevant to its use in sewer environments and other outdoor applications:

- Thermal Resistance: Polyurea coatings are known for their stability across a wide range of temperatures, making them suitable for structures exposed to seasonal temperature fluctuations. The material maintains its flexibility and adhesion at both high and low temperatures, reducing the risk of cracking or delamination.
- Waterproof Performance: Polyurea’s hydrophobic nature ensures excellent waterproofing, which is critical in protecting the underlying concrete from further moisture ingress. This property is particularly valuable in sewer applications, where prolonged exposure to aggressive, moisture-rich environments is common.
- Material Aging under Solar Irradiation: While sewer environments typically limit UV exposure, outdoor manholes and similar infrastructure may be subjected to solar radiation. Polyurea demonstrates good resistance to UV-induced degradation, maintaining its protective and mechanical properties over time. However, additional coatings or UV stabilizers may further enhance its performance in highly exposed environments.

These attributes enhance the longevity of the repair system, especially when combined with the adaptability of the polyurethane foam layer, which varies in thickness to address localized damage. While this study focuses on mechanical performance, these broader material properties underscore the versatility and practicality of the three-layer system.

The results emphasize the necessity of tailoring repair systems to the degree and variability of degradation. The sectional homogenization approach efficiently captures the average mechanical behavior of repaired sections but is limited in accounting for localized deviations in damage or material performance. This limitation suggests a need for further refinement, such as incorporating localized property variations into the model or combining homogenization with higher-fidelity simulations in critical areas.

The repair system’s practical applications are broad, particularly for non-critical infrastructure where full restoration of load-bearing capacity is not essential. However, for critical applications, supplementary reinforcement—such as embedded fibers or mesh—could enhance bending and shear resistance, addressing the stiffness limitations highlighted in this study.

In summary, the three-layer repair method offers significant improvements in structural performance, chemical resistance, and durability. While the study highlights the limitations of restoring full mechanical properties, the adaptability of the polyurethane foam layer and the robust protective qualities of polyurea make this system a practical and versatile solution for extending the lifespan of degraded concrete structures. Future research could focus on experimental validation, optimization of material properties, and integration of advanced reinforcement strategies to further improve the system’s performance.

## 5. Conclusions

This study explored the effectiveness of a three-layer protective and reinforcing coating in restoring the structural integrity of heavily corroded concrete manholes. Through a combination of 3D scanning, homogenization, and finite element modeling, we assessed the repaired structure’s load-bearing capacity and stiffness in comparison to both the original and degraded states. The findings demonstrate that while the repair method significantly improves the structural performance, it does not fully restore the mechanical properties of the original undamaged concrete, especially in cases of extensive corrosion.

The use of an effective thickness through homogenization proved essential for accurate analysis, allowing the repaired structure to be represented as a single layer for efficient modeling. This approach enabled us to capture the mechanical response under both compressive and bending states, confirming that homogenization, coupled with segmentation, is critical for reliable performance assessment. However, residual reductions in membrane, bending, and shear stiffness were evident, even after repair, with the degree of residual loss correlating to the extent of initial corrosion.

The limitations of the three-layer repair method lie primarily in its material composition and design focus. While the polyurethane foam layer is effective in filling voids, its lower intrinsic stiffness compared to concrete restricts its contribution to structural load-bearing capacity. Furthermore, the primary role of the polyurea layers as protective barriers means that they contribute minimally to mechanical reinforcement. This repair approach, therefore, may be more suitable for applications where chemical protection and extension of service life are primary goals, rather than full structural restoration.

This study demonstrates that sectional homogenization effectively reduces computational demands while providing accurate assessments of the mechanical behavior of repaired concrete manholes. Key findings indicate that: (a) stiffness recovery varies by degradation level, with bending stiffness reaching up to 76% restoration in heavily corroded segments, and (b) the limitations of polyurethane foam as a core reinforcement material highlight the need for integrating higher-stiffness components or tailored repair strategies. Future research should prioritize experimental validation and explore the integration of fiber-reinforced layers or other innovative materials to improve repair outcomes in critical infrastructure.

Overall, the three-layer coating offers a practical solution for prolonging the lifespan of corroded infrastructure, but additional research into higher-stiffness materials and alternative reinforcement techniques is warranted to enhance its structural efficacy. Future work should consider composite materials, targeted reinforcement in critical areas, and more customized application methods to improve the repair’s ability to restore load-bearing capacity fully. This study underscores the importance of selecting repair methods based on specific structural needs, highlighting that while current techniques offer valuable protection, further innovation is needed to meet the demands of critical infrastructure fully.

## Figures and Tables

**Table 1:** Changes in **A**, **D**, and **R** stiffness values for the least corroded segment with a volume loss of ΔV=−10.19%, comparing original, corroded, and repaired conditions.

Changes in Stiffness	Deteriorated vs. Initial	Repaired vs. Initial	Repaired vs. Deteriorated
ΔA **[%]**	−10.19	−8.45	1.94
ΔD **[%]**	−22.78	−7.93	19.23
ΔR **[%]**	−10.19	−7.99	2.45

**Table 2:** Effective material parameters for the homogenized repaired section with an effective thickness of $t_{eff}=111.27$ mm, showing both effective and total thickness values.

Effective Parameters	From D Matrix	From A Matrix	From R Matrix
$E_{11}$$/E_{22}$ [MPa]	30,993 (5337)	30,993 (17,243)	-
$\nu_{12}$ [-]	0.24999	0.24995	-
$G_{12}$ [MPa]	11,623 (2002)	11,622 (6466)	-
$G_{13}$$/G_{23}$ [MPa]	-	-	9685 (5387)

**Table 3:** Changes in **A**, **D**, and **R** stiffness values for a segment with significant corrosion (ΔV=−38.27%), comparing original, corroded, and repaired states.

Changes in Stiffness	Deteriorated vs. Initial	Repaired vs. Initial	Repaired vs. Deteriorated
ΔA **[%]**	−38.26	−35.82	3.96
ΔD **[%]**	−71.93	−50.60	76.00
ΔR **[%]**	−38.26	−35.17	5.01

**Table 4:** Effective material parameters for the heavily corroded and repaired segment with an effective thickness of $t_{eff}=80.92$ mm, including values for both effective and total thicknesses.

Effective Parameters	From D Matrix	From A Matrix	From R Matrix
$E_{11}$$/E_{22}$ [MPa]	29,296 (1940)	29,298 (11,853)	-
$\nu_{12}$ [-]	0.24999	0.24978	-
$G_{12}$ [MPa]	10,989 (727)	10,987 (4445)	-
$G_{13}$$/G_{23}$ [MPa]	-	-	9156 (3704)

## Data Availability

The original contributions presented in the study are included in the article, further inquiries can be directed to the corresponding author.

## Appendix A

Table A1 presents the **A**, **D**, and **R** matrices for the original, undamaged material. In this case, the concrete wall has an initial thickness of 120 mm, and the values reflect the baseline mechanical properties of the manhole before any degradation.

**Table A1:** Parameters in **A**, **D**, and **R** matrices for the original undamaged concrete material with a wall thickness of 120 mm.

A Matrix	D Matrix	R Matrix
$\left[\frac{N}{mm^{2}}\cdot mm\right]\times 10^{6}$	$\left[\frac{N}{mm^{2}}\cdot mm^{3}\right]\times 10^{9}$	$\left[\frac{N}{mm^{2}}\cdot mm\right]\times 10^{6}$
$\left[\begin{array}{ccc} 3.840 & 0.960 & 0\\ 0.960 & 3.840 & 0\\ 0 & 0 & 1.440 \end{array}\right]$	$\left[\begin{array}{ccc} 4.608 & 1.152 & 0\\ 1.152 & 4.608 & 0\\ 0 & 0 & 1.728 \end{array}\right]$	$\left[\begin{array}{cc} 1.000 & 0\\ 0 & 1.000 \end{array}\right]$

Table A2 shows the **A**, **D**, and **R** matrices for a degraded manhole segment that has been repaired using the three-layer protective and reinforcing coating described above. These values indicate the modified stiffness properties of the structure post-repair, compensating for material loss due to corrosion.

**Table A2:** Parameters in **A**, **D**, and **R** matrices for the manhole segment after corrosion and subsequent repair using the three-layer protective coating.

A Matrix	D Matrix	R Matrix
$\left[\frac{N}{mm^{2}}\cdot mm\right]\times 10^{6}$	$\left[\frac{N}{mm^{2}}\cdot mm^{3}\right]\times 10^{9}$	$\left[\frac{N}{mm^{2}}\cdot mm\right]\times 10^{6}$
$\left[\begin{array}{ccc} 3.449 & 0.862 & 0\\ 0.862 & 3.449 & 0\\ 0 & 0 & 1.293 \end{array}\right]$	$\left[\begin{array}{ccc} 3.558 & 0.889 & 0\\ 0.889 & 3.558 & 0\\ 0 & 0 & 1.334 \end{array}\right]$	$\left[\begin{array}{cc} 0.898 & 0\\ 0 & 0.898 \end{array}\right]$

Table A3 shows the **A**, **D**, and **R** matrices for a heavily degraded manhole segment.

**Table A3:** Parameters in **A**, **D**, and **R** matrices for a heavily corroded manhole segment with 38.27% volume loss and subsequent repair.

A Matrix	D Matrix	R Matrix
$\left[\frac{N}{mm^{2}}\cdot mm\right]\times 10^{6}$	$\left[\frac{N}{mm^{2}}\cdot mm^{3}\right]\times 10^{9}$	$\left[\frac{N}{mm^{2}}\cdot mm\right]\times 10^{6}$
$\left[\begin{array}{ccc} 2.371 & 0.593 & 0\\ 0.593 & 2.371 & 0\\ 0 & 0 & 0.889 \end{array}\right]$	$\left[\begin{array}{ccc} 1.293 & 0.323 & 0\\ 0.323 & 1.293 & 0\\ 0 & 0 & 0.485 \end{array}\right]$	$\left[\begin{array}{cc} 0.617 & 0\\ 0 & 0.617 \end{array}\right]$
